# Evaluating the concordance between sequencing, imputation and microarray genotype calls in the GAW18 data

**DOI:** 10.1186/1753-6561-8-S1-S22

**Published:** 2014-06-17

**Authors:** Ally Rogers, Andrew Beck, Nathan L Tintle

**Affiliations:** 1 Department of Mathematics, Statistics and Computer Science, Dordt College, Sioux Center, IA 51250, USA; 2 Department of Mathematics, Loyola University Chicago, Chicago, IL 60660, USA

## Abstract

Genotype errors are well known to increase type I errors and/or decrease power in related tests of genotype-phenotype association, depending on whether the genotype error mechanism is associated with the phenotype. These relationships hold for both single and multimarker tests of genotype-phenotype association. To assess the potential for genotype errors in Genetic Analysis Workshop 18 (GAW18) data, where no gold standard genotype calls are available, we explored concordance rates between sequencing, imputation, and microarray genotype calls. Our analysis shows that missing data rates for sequenced individuals are high and that there is a modest amount of called genotype discordance between the 2 platforms, with discordance most common for lower minor allele frequency (MAF) single-nucleotide polymorphisms (SNPs). Some evidence for discordance rates that were different between phenotypes was observed, and we identified a number of cases where different technologies identified different bases at the variant site. Type I errors and power loss is possible as a result of missing genotypes and errors in called genotypes in downstream analysis of GAW18 data.

## Background

Over the past decade, a large body of literature has been amassed related to genotype errors for SNP microarrays. We now have a clear understanding of the prevalence of such errors and of many potential sources of the errors, as well as an understanding of the downstream implications of genotype errors on the type I error rate and power of related single SNP tests of genotype-phenotype association [1]. In particular, nondifferential genotyping errors, that is, errors that are the result of a random process unrelated to the phenotype, decrease power [2-4]. However, differential genotyping errors, errors that occur according to different random processes according to the value of the phenotype, may inflate the type I error rate [5,6]. Additional work has confirmed that similar results hold for analysis of imputed genotypes using standard single-marker tests of genotype-phenotype association [7].

With the advent of next-generation sequencing (NGS), multimarker analysis methods have increased in popularity. Recent papers demonstrate similar results (i.e., decreased power and increased type I error for nondifferential and differential genotyping errors) are true for multimarker tests as well. In particular, for collapsing tests [e.g., [8-10]], the effects of both differential and nondifferential genotyping errors can be exacerbated by the cumulative nature of genotyping errors across a set of markers [11,12]. The relationship for particular collapsing tests is anticipated to hold for the larger set of all collapsing (burden) and variance components tests based on structural similarities in these classes of tests [13]. To date, large error rates have been observed for sequence data [14-16], much larger than were typical in the early days of SNP microarrays [17]. Thus, there is the potential for substantial power loss and inflated type I error for multimarker tests involving NGS data.

For the typical researcher, it is often costly and impractical to invest in large-scale quality control studies to obtain study-specific estimates of genotype reliability. However, as was seen in the GAW18 data, it is reasonable to think that as more and more studies sequence existing samples, a typical quality control approach may involve evaluating the concordance between genotypes obtained on the samples using SNP microarrays with genotypes obtained using the new NGS technology. We conducted our analysis using sequencing data (measured with NGS technology or through imputation) and SNP microarray data. After evaluating the overall concordance levels between genotype calls, we evaluated which types of discordance are most common and the potential for concordance rates, which are related to the phenotype.

## Methods

We used the following procedure to evaluate the concordance of sequence and microarray data. First, we considered all SNPs for which both sequence and microarray data were available in the distributed GAW18 files by matching SNP identification (rs) numbers. Prior to our analysis, each set of data went through separate data cleaning pipelines, which included cleaning observed mendelian errors within the pedigrees for both the sequence and microarray data and which are described in detail elsewhere [18]. This yielded a preliminary data set containing 297,197 SNPs. After eliminating SNPs for which the major and minor alleles present at the variant site differed between the 2 technologies (56,741 SNPs), the resulting final analysis data set consisted of 240,456 SNPs, spread across all odd-numbered autosomes. Even-numbered autosomes and sex chromosomes were not part of the GAW18 data release. Next, for each of the 240,456 SNPs in the analysis data set, we identified and recorded both the genome-wide association studies (GWAS) and NGS genotypes (including missing) for each of 959 people for whom both GWAS and NGS data was available. The 959 people include 464 individuals who were actually sequenced and 495 individuals for whom sequence data was imputed using MaCH as described elsewhere [18].

### Statistical analysis

For each SNP in the analysis we computed a variety of statistics evaluating the concordance between genotype calls on the 3 different platforms (NGS, imputed, and SNP microarray). We started by counting the overall number of concordant and discordant genotypes for sequenced and microarray data. There are 16 possibilities for each individual-SNP combination: AA-AA, AA-AB, AA-BB, AA-XX, AB-AA, AB-AB, AB-BB, AB-XX, BB-AA, BB-AB, BB-BB, BB-XX, XX-AA, XX-AB, XX-BB, and XX-XX, where *i-j *indicates that the individual is identified as genotype *i *for sequence data and genotype *j *for microarray data. (Note that we use "A" to represent the reference allele for the NGS technology, "B" to represent the nonreference allele for the NGS technology, and × to represent missing throughout this article.)

In addition to overall concordance, concordance rates were computed conditional on the observed genotype for the microarray technology. Concordance rates were also computed for individuals with different phenotype groups (males vs. females; hypertensive [systolic blood pressure >140 mm Hg or diastolic blood pressure >90 mm Hg at any of 4 exams]; vs. nonhypertensive smokers [self-identified at any of 4 waves] vs. nonsmokers). T-tests were used to compare average concordance rates between technologies and between phenotypes across the set of all SNPs.

## Results

Table 1 cross-classifies all 240,456 SNPs for which both SNP microarray and sequence data are available, and which met our initial screening criteria (see Methods for details). Across the 230,597,304 (240,456 SNPs × 959 individuals) possible genotype calls, there are more than 500,00 discordant genotypes (both technologies call a genotype, and the genotypes are different), and more than 5 million genotypes that are missing on at least 1 of the 2 platforms. This means that the overall proportion of discordant genotypes (including missing) is 2.63%, while the proportion of discordant called genotypes is 0.23%.

**Table 1:** Cross-classification of results summed over all SNPs and individuals

Sequence genotype^1 ^	Microarray genotype				Total
		
	AA	AB	BB	Missing (XX)	
AA	117,284,236	58,271	1,309	2,554	117,346,370
AB	101,015	65,584,521	29,302	8,970	65,723,808
BB	6,844	339,856	41,656,995	24,361	42,028,056
Missing (XX)	3,009,304	1,506,621	977,234	5,911	5,499,070
Total	120,401,399	67,489,269	42,664,840	41,796	230,597,304

To gain a better understanding of the distribution of the discordant genotypes noted in Table 1, we computed conditional concordance rates (Table 2). In particular, we examined the probabilities that the sequence technology yields each different genotype (or missing) conditional on the genotype identified by the microarray technology. Table 2 provides separate conditional concordance rates for NGS and imputed genotypes.

**Table 2:** Conditional concordance rates (conditional on microarray genotype; SE in parentheses)

Sequence genotype^1^		Microarray genotype			
	
		AA	AB	BB	Missing (XX)
AA	NGS	0.998 (0.03)	0.003 (0.03)	0.004 (0.06)	0.52 (0.36)
	Imp	0.998 (0.02)	0.02 (0.11)	0.0006 (0.02)	0.44 (0.46)

AB	NGS	0.0009 (0.02)	0.996 (0.04)	0.02 (0.08)	0.30 (0.24)
	Imp	0.002 (0.02)	0.980 (0.12)	0.006 (0.06)	0.38 (0.43)

BB	NGS	0.0007 (0.02)	0.0006 (0.02)	0.98 (0.10)	0.19 (0.28)
	Imp	8 × 10^−5 ^(0.008)	0.003 (0.05)	0.993 (0.07)	0.18 (0.35)

Missing (XX)	NGS	8 × 10^−5 ^(0.005)	0.0006 (0.02)	0.0007 (0.01)	0.001 (0.02)
	Imp	8 × 10^−5 ^(0.005)	0.0003 (0.009)	0.0004 (0.01)	0.007 (0.07)

As Table 2 shows, in a number of cases, the average concordance rates are substantially different between the 2 sequencing technologies. In fact, except for AA given AA, XX (missing) given AA and BB given XX (missing), all p-values from t-tests comparing the average rates between the 2 technologies are less than 2 × 10^−16^. When the microarray platform identifies the genotype as a nonreference allele homozygote, imputed sequence data shows higher concordance than NGS data. When the microarray identifies the genotype as a homozygote reference allele, rates of discordance for the homozygous nonreference allele genotype are also higher for imputed data compared to NGS genotypes. However, when the microarray platform calls the genotype a heterozygote, the NGS sequence genotypes are more concordant, as is the case when the microarray platform identifies the genotype as a reference allele homozygote and the discordance rates for the heterozygote genotype are compared. When the SNP microarray genotype is missing, the sequence data often identifies at least 1 reference allele at the site. There is also a strong association between MAF and concordance rates. In particular, SNPs with lower MAF have substantially lower concordance between platforms than do SNPs with larger MAFs (detailed results not shown).

Finally, Table 3 illustrates an analysis comparing the average conditional concordance rates for hypertensive individuals compared to nonhypertensive individuals. The strongest evidence for significant differences (*p *<2 × 10^−16^) in average conditional concordance were observed between hypertensive and nonhypertensives for sequence AA or BB given GWAS AA, sequence AA or AB given GWAS AB, and sequence AA, AB, or BB given GWAS BB. Some evidence of differential average conditional concordance rates between males and females and smokers and nonsmokers also exists (detailed results not shown).

**Table 3:** Conditional concordance rates of hypertensive vs. nonhypertensive individuals (conditional on microarray; SE in parentheses)

Sequence genotype		Microarray genotype			
	
		AA	AB	BB	Missing (XX)
AA	Hypertensive	0.998 (0.03)	0.009 (0.07)	0.005 (0.07)	0.51 (0.43)
	Nonhypertensive	0.999 (0.02)	0.010 (0.07)	0.0004 (0.02)	0.51 (0.37)

AB	Hypertensive	0.001 (0.02)	0.990 (0.08)	0.013 (0.07)	0.30 (0.36)
	Nonhypertensive	0.001 (0.02)	0.987 (0.08)	0.011 (0.07)	0.30 (0.26)

BB	Hypertensive	0.0009 (0.03)	0.002 (0.03)	0.982 (0.10)	0.19 (0.33)
	Nonhypertensive	4 × 10^−5 ^(0.005)	0.002 (0.04)	0.989 (0.07)	018 (0.28)

Missing (XX)	Hypertensive	7 × 10^−5 ^(0.005)	0.0004 (0.009)	0.0004 (0.01)	0.001 (0.02)
	Nonhypertensive	7 × 10^−5 ^(0.004)	0.0005 (0.01)	0.0005 (0.01)	0.002 (0.02)

## Discussion

Although most genotypes are the same for both technologies, there are still substantial numbers of discordant genotype pairs. The most common type of discordance comes from missing genotypes on the sequence technology, which occurred most frequently when the microarray technology identified at least 1 reference allele at the variant site. Power loss will occur when genotypes are not called, and so using sequence technology genotypes will, when analyzing single SNPs or sets of SNPs considered in this analysis, yield lower power overall than using microarray genotypes. We note, however, that overall power may still be higher when using sequenced genotypes as our analysis necessarily precludes the inclusion of less common SNPs, which are not measured by the microarray technology.

Although discordance between called genotypes is less common than with missing genotypes, the amount of discordance (500,000 discordant genotypes; 0.23% overall discordance rate) is still notable. Of particular note are the high proportions of heterozygote (microarray) to nonreference allele homozygote and reference allele homozygote (microarray) to heterozygote (sequence) discrepancies; more than 80% of all called genotype discordance is from these 2 types of discrepancies. The conditional discordance rates in Table 2 suggest that the majority of the discordance occurs in the imputed sequence (not the NGS sequence) data. Given that the NGS data comes from 60× coverage, the microarray genotype calling pipeline is well established, and the imputation procedure used in the GAW18 data is both novel and complex, we conclude it likely that aspects of the imputation procedure is what yielded the majority of observed discordant genotypes.

It is reasonable to view the conditional discordant genotype rates in Table 2 as conservative estimates of the genotype error rate because, if the technologies are applied independently, the vast majority of genotype errors of each technology will appear in a discordant genotype pair. However, if genotype errors are correlated between the 2 technologies (eg, at a particular variant site, similar samples are prone to error on both technologies), using the conditional discordant rate as an estimate of the genotype error rate may be substantially lower than the true genotype error rate.

As documented for single-marker tests, genotyping errors from the major homozygote to the minor homozygote are the most costly (in terms of power loss) [2,4,19]. Recently, Powers et al [11] documented potentially large declines in statistical power for collapsing tests in case-control designs, when genotype errors (particularly from more common to less common genotypes) are present, as is the case here. For example, with genotype error rates of 0.2% to 0.5% for more common to less common genotypes (as estimated in Table 2), power loss between 2% and 5% for most collapsing tests will occur. Genotype error rates of up to 2% from less common to more common genotypes have only modest impact on power (<0.5% decline). Because these results were for case-control studies, further research is needed to demonstrate similar effects of genotyping errors on family based collapsing tests. These papers [2,4,11,19] also found that power loss increases as the MAF decreases; because discordance was larger for lower MAF SNPs, power loss will be larger for lower MAF SNPs in GAW18 data. Furthermore, our analysis only considered SNPs with MAF above 5%, as rarer SNPs were not genotyped using the microarray technology. If the trend we observed continues, these SNPs may have even larger error rates and, hence, even more dramatic power loss.

These problems are further compounded when we consider that there was some evidence of differential discordance between phenotypes. Differential discordance can lead to inflated type I errors; for example, in line with our observation, for collapsing tests, error rates of 0.2% in cases and 0.1% in controls (or vice versa) can inflate the type I error rate from 5% to between 15% and 25% for most tests [5,6,12]. Although quality-control approaches (eg, Q-Q plots) can detect large-scale type I error deviations, isolated differential genotyping errors may escape typical quality control and manifest themselves as false positives. To minimize the effect of differential genotyping errors, random assignment of subjects across genotyping laboratories, laboratory assistants and plates should always be practiced. Although the precise cause of the modest differential genotyping errors observed here is unknown, it may be a result of familial aggregation of hypertension and nonrandom assignment of family members to sequencing runs, imputation quality differences between families, or other unmeasured covariates.

We note that our analysis did not consider many of the most egregious inconsistencies: namely, 56,741 SNPs where the allele calls were different. Further analysis is needed to identify whether the allele call differences yield substantially different genotype distributions in the cases and controls between the 2 technologies, or it is simply a matter of exchanging one base for another in the calling algorithms (eg, reverse and forward strand differences between the 2 technologies).

It is likely that, increasingly, sequence and microarray data will be available for the same sample as was the case here. In addition to providing an opportunity to empirically evaluate data quality, as was done here, discordant genotypes present an opportunity to utilize better quality data in downstream analyses. Recently, we showed that, under modest assumptions of independence of the 2 independent genotype mechanisms, a 50-50 weighting strategy of the 2 discordant genotypes should be used in analyses of phenotype-genotype association [20]. Thus, a reasonable choice for the genotype to be analyzed in cases of discordance is the dosage, where dosage = 0.5 is used for major homozygote-heterozygote discordant pairs and dosage = 1.5 is used for heterozygote-minor homozygote pairs. Further work is needed to confirm the optimality of this result for multimarker rare variant tests of association.

## Conclusions

Despite sophisticated data-cleaning pipelines for all 3 technologies, a noticeable number of discordant genotypes remain in the GAW18 data. It is encouraging that the majority of discordant genotypes were identified as missing by one of the technologies; however, a substantial number of discordant called genotypes were still observed. Although the amounts and types of discordance observed here will likely lead to power loss and/or type I errors in downstream analysis, further research is needed to understand the impact of errors on tests of association in family based studies using sets of markers. However, it is reasonable to expect that errors and missing data will likely impact the type I and/or power for family based tests as they do for case-control tests.

## Competing interests

The authors declare that they have no competing interests.

## Authors' contributions

NT designed the overall study, AB created the necessary datasets, AR and NT conducted the statistical analysis, and NT drafted the manuscript. All authors read and approved the final manuscript.

## References

[B1] Gordon D Finch SJ Factors affecting statistical power in the detection of genetic association J Clin Invest 2005 115 1408 1418 10.1172/JCI24756 15931375 PMC1137002

[B2] Ahn K Haynes C Kim W Fleur RS Gordon D Finch SJ The effects of SNP genotyping errors on the power of the Cochran-Armitage linear trend test for case/control association studies Ann Hum Genet 2007 71 249 261 10.1111/j.1469-1809.2006.00318.x 17096677

[B3] Gordon D Finch SJ Nothnagel M Ott J Power and sample size calculations for case-control genetic association tests when errors are present: application to single nucleotide polymorphisms Hum Hered 2002 54 22 33 10.1159/000066696 12446984

[B4] Kang SJ Gordon D Finch SJ What SNP genotyping errors are most costly for genetic association studies? Genet Epidemiol 2004 26 132 141 10.1002/gepi.10301 14748013

[B5] Ahn K Gordon D Finch SJ Increase of rejection rate in case-control studies with differential genotyping error rates Stat Appl Genet Mol Biol 2009 8 25 19492983 10.2202/1544-6115.1429

[B6] Moskvina V Craddock N Holmans P Owen MJ O'Donovan MC Effects of differential genotyping error rate on the type I error probability of case-control studies Hum Hered 2006 61 55 64 10.1159/000092553 16612103

[B7] Huang L Wang C Rosenberg NA The relationship between imputation error and statistical power in genetic association studies in diverse populations Am J Hum Genet 2009 85 692 698 10.1016/j.ajhg.2009.09.017 19853241 PMC2775841

[B8] Li B Leal SM Methods for detecting associations with rare variants for common diseases: application to analysis of sequence data Am J Hum Genet 2008 83 311 321 10.1016/j.ajhg.2008.06.024 18691683 PMC2842185

[B9] Madsen BE Browning SR A group-wise association test for rare mutations using a weighted sum statistic PLoS Genet 2009 5 e1000384 10.1371/journal.pgen.1000384 19214210 PMC2633048

[B10] Morris AP Zeggini E An evaluation of statistical approaches to rare variant analysis in genetic association studies Genet Epidemiol 2010 34 188 193 10.1002/gepi.20450 19810025 PMC2962811

[B11] Powers S Gopalakrishnan S Tintle NL Assessing the impact of non-differential genotyping errors on rare variant tests of association Hum Hered 2011 72 153 160 10.1159/000332222 22004945 PMC3214826

[B12] Mayer-Jochimsen M Tintle NL Assessing the impact of differential genotyping errors on rare variant tests of association PLoS One 2013 8 e56626 10.1371/journal.pone.0056626 23472072 PMC3589406

[B13] Liu K Fast S Zawistowski M Tintle NL A geometric framework for the evaluation of rare variant tests of association Genet Epidemiol 2013 37 345 357 10.1002/gepi.21722 23526307 PMC3718063

[B14] Awadalla P Gauthier J Myers RA Casals F Hamdan FF Griffing AR Côté M Henrion E Spiegelman D Tarabeux J Direct measure of the de novo mutation rate in autism and schizophrenia cohorts Am J Hum Genet 2010 87 316 324 10.1016/j.ajhg.2010.07.019 20797689 PMC2933353

[B15] Ilie L Fazeyeli F Ille S HiTEC: accurate error correction in high-throughput sequencing data Bioinformatics 2011 27 295 302 10.1093/bioinformatics/btq653 21115437

[B16] Nielsen R Paul JS Albrechtsen A Song YS Genotype and SNP calling from next-generation sequencing data Nat Rev Genet 2011 12 443 451 10.1038/nrg2986 21587300 PMC3593722

[B17] Tintle NL Ahn K Mendell NR Gordon D Finch SJ Characteristics of replicated single-nucleotide polymorphism genotypes from COGA: Affymetrix and Center for Inherited Disease Research BMC Genet 2005 6 Suppl 1 S154 10.1186/1471-2156-6-S1-S154 16451615 PMC1866780

[B18] Genetic Analysis Workshop 18 2012 http://www.gaworkshop.org GAW18_Data_Description.pdf. Distributed with workshop materials. July

[B19] Kang SJ Finch SJ Haynes C Gordon D Quantifying the percent increase in minimum sample size necessary for SNP genotyping errors in genetic model-based association studies Hum Hered 2004 58 139 144 10.1159/000083540 15812170

[B20] Tintle NL Gordon D McMahon FJ Finch SJ Using duplicate genotyped data in genetic analyses: testing association and estimating error rates Stat Appl Genet Mol Biol 2007 6 Article 4 17402919 10.2202/1544-6115.1251

